# Localized measurements of water potential reveal large loss of conductance in living tissues of maize leaves

**DOI:** 10.1093/plphys/kiad679

**Published:** 2023-12-21

**Authors:** Piyush Jain, Annika E Huber, Fulton E Rockwell, Sabyasachi Sen, Noel Michele Holbrook, Abraham D Stroock

**Affiliations:** Sibley School of Mechanical and Aerospace Engineering, Cornell University, Ithaca, NY 14853, USA; Smith School of Chemical and Biomolecular Engineering, Cornell University, Ithaca, NY 14853, USA; Department of Organismic and Evolutionary Biology, Harvard University, Cambridge, MA 02138, USA; Sibley School of Mechanical and Aerospace Engineering, Cornell University, Ithaca, NY 14853, USA; Department of Organismic and Evolutionary Biology, Harvard University, Cambridge, MA 02138, USA; Smith School of Chemical and Biomolecular Engineering, Cornell University, Ithaca, NY 14853, USA; School of Integrative Plant Science, Cornell University, Ithaca, NY 14853, USA; Kavli Institute at Cornell for Nanoscale Science, Cornell University, Ithaca, NY 14853, USA

## Abstract

The water status of the living tissue in leaves between the xylem and stomata (outside xylem zone (OXZ) plays a critical role in plant function and global mass and energy balance but has remained largely inaccessible. We resolve the local water relations of OXZ tissue using a nanogel reporter of water potential (ψ), AquaDust, that enables an in situ, nondestructive measurement of both *ψ* of xylem and highly localized *ψ* at the terminus of transpiration in the OXZ. Working in maize (*Zea mays* L.), these localized measurements reveal gradients in the OXZ that are several folds larger than those based on conventional methods and values of *ψ* in the mesophyll apoplast well below the macroscopic turgor loss potential. We find a strong loss of hydraulic conductance in both the bundle sheath and the mesophyll with decreasing xylem potential but not with evaporative demand. Our measurements suggest the OXZ plays an active role in regulating the transpiration path, and our methods provide the means to study this phenomenon.

## Introduction

**Figure 1.**
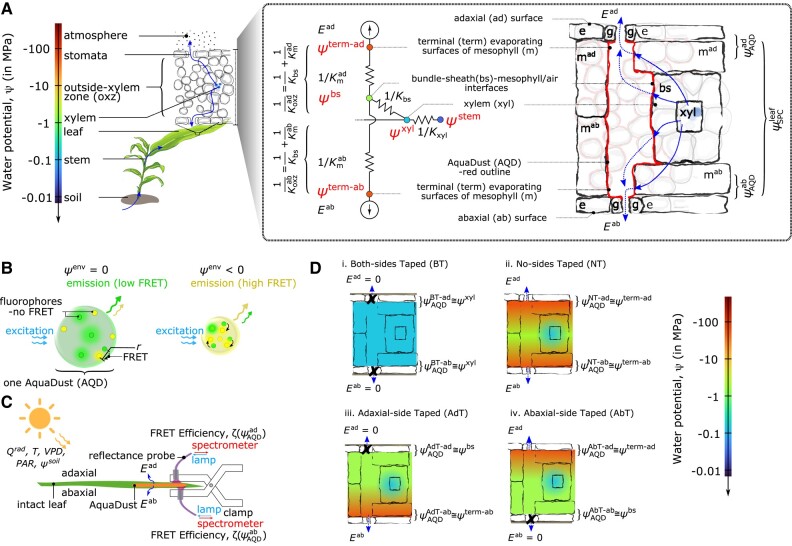
Dissection of water relations of OXZ with AquaDust. **A)** Schematic representation of soil-plant-atmosphere continuum along gradient of water potential (ψ) and corresponding hydraulic circuit. Expanded representation of a leaf cross-section shows the different cell types and pathways for water movement in the OXZ. The hydraulic circuit (left) presents a simplified representation of these water paths to the adaxial (top) and abaxial (bottom) surfaces of the leaf: starting in the xylem at water potential $\psi^{\mathrm{xyl}}$, water passes through the bundle-sheath conductance $\left(K_{\mathrm{bs}}\right)$ and the mesophyll conductance $\left(K_{m}\right)$ to reach the terminal sites of evaporation in the sub-stomatal cavity, at water potential $\psi^{\mathrm{term}}$; water vapor then diffuses through stomata into the atmosphere with transpiration rate *E*. The expanded representation of a leaf cross-section (right) shows individual cells (gray outlines) and a coarser depiction of the different tissue compartments (black outlines) and pathways for water movement (blue arrows) in the OXZ; this depiction of compartments is commensurate with simplified circuit representation on the left. The nanoparticle reporter, AquaDust (Jain et al. 2021) coats the apoplastic surfaces of the mesophyll at the interface with the intercellular air spaces, as shown with red lines around the tissue compartments and grayed-out red lines around the individual cells. Its fluorescence signal reports a value of water potential from a narrow depth beneath the epidermis ($\psi_{\mathrm{AQD}}$—as labeled), that should approximate the values of $\psi^{\mathrm{term}\;}$. In contrast, the Scholander pressure chamber provides a tissue-averaged value of water potential ($\psi_{\mathrm{SPC}}^{\mathrm{leaf}\;}$—as labeled). **B)** Schematic diagrams showing mechanism of AquaDust nanoreporters based on FRET between pairs of dyes (green and yellow circles) in a hydrogel nanoparticle: the swollen, “wet” state when water potential in its local environment $\psi^{\mathrm{env}}=0$ (i.e. saturated condition), results in low FRET between donor (green circles) and acceptor (yellow circles) dye (left); and the shrunken, “dry” state when $\psi^{\mathrm{env}\;}<0$ (i.e. stressed condition) results in high FRET between fluorophores, thereby altering the emission spectra (right). Details on synthesis, characterization, and calibration of AquaDust are described by Jain et al. (2021). **C)** Schematic representation of a leaf undergoing transpiration through adaxial ($E^{\mathrm{ad}}$) and abaxial ($E^{\mathrm{ab}}$) surfaces of the leaf in a dynamic environment as a function of solar thermal radiation $\left(Q^{\mathrm{rad}}\right)$ and photosynthetically active radiation (PAR), temperature (T), VPD, and soil water potential $\left(\psi^{\mathrm{soil}}\right)$. Zones on the leaves infiltrated with AquaDust (orange patches on diagram) serve as reporters of the local water potential at the terminus of the adaxial $\left(\psi_{\mathrm{AQD}}^{\mathrm{ad}}≅\psi^{\mathrm{term}\text{-}\mathrm{ad}}\right)$ and abaxial $\left(\psi_{\mathrm{AQD}}^{\mathrm{ab}}≅\psi^{\mathrm{term}\text{-}\mathrm{ab}}\right)$ sides, captured using a short (∼30s), minimally invasive measurement of FRET efficiency ($\zeta (\psi^{\mathrm{ab}\;})$ and $\zeta (\psi^{\mathrm{ad}\;})$ respectively) using a fiber probe positioned to collect fluorescence from the adaxial and abaxial leaf surfaces, as shown. **D)** Scenarios used to manipulate the local, through-thickness gradients of water potential in leaf with selective blocking of the transpiration stream with a transparent tape: (i) both sides obstructed (BT), (ii) neither side obstructed (NT), and one side obstructed ((iii) adaxial taped (AdT) or (iv) abaxial taped (AbT)). These manipulations allow the water potential measured by AquaDust to provide values of water potential at different locations in the leaf by local equilibrium, as indicated with approximate equations.

Terrestrial plants play a dominant role in mediating the exchange of water, carbon dioxide (CO_2_), and energy between the Earth's surface and the atmosphere. Transpiration from plants accounts for ∼64% of the water flux from the land surface into the atmosphere (Good et al. 2015) and is the dominant driver of the human use of fresh water through irrigated agriculture (Food and Agriculture Organisation 2017). In coupling the soil to an unsaturated atmosphere, plants conduct water across a massive range in water potential, *ψ*, from near zero in the soil to ∼−100MPa in the atmosphere (Fig. 1A). A long-standing consensus holds that the majority of this drop in potential occurs across the stomata, in the vapor phase between the interior of the leaf and the atmosphere (Nobel 1999). Plants are assumed to achieve this control of tissue water potential by regulating stomata to maintain the mesophyll, the living tissue in the leaves, within a few MPa of leaf xylem water potential $\left(\psi^{\mathrm{xyl}}\right)$, and, as a result, we expect internal mesophyll conductance to be high to keep this critical tissue close to xylem water status (Van Den Honert 1948). But, recent work suggests that passage of the transpiration stream across the outside xylem zone (OXZ), through the bundle sheath and mesophyll to terminal sites of evaporation (Fig. 1A), can also mediate a large drop in potential; further, this drop can vary based on changes in the conductance of the OXZ $\left(K_{\mathrm{oxz}}\right)$ with the water status of the leaf and evaporative demand (Scoffoni et al. 2017; Cernusak et al. 2018; Wong et al. 2022). Nonetheless, we lack information on the constitutive properties of the OXZ and understanding of their regulation—active and passive—as a function of environmental and biological stresses (Buckley 2017, 2019; Rockwell and Holbrook 2017). This knowledge gap hinders scientific progress on critical outstanding questions about drought stress physiology in agricultural (Corso et al. 2020; Wu et al. 2021) and ecophysiological (Buckley 2015, 2019) contexts and our ability to design management strategies and cultivars that are efficient and resilient with respect to water-use.

Early studies using pressure probes (Sheriff 1982; Shackel and Brinckmann 1985; Shackel 1987; Frensch and Schulze 1988; Nonami and Schulze 1989) allowed for noninvasive interrogation of OXZ water relations; however, reproducing these methods proved challenging and further use of them has not been reported. More recent studies of leaf water relations have combined whole-leaf measurements of water potential on excised tissue with the Scholander pressure chamber (SPC) and flow measurements or gas exchange to assess OXZ conductance $\left(K_{\mathrm{oxz}}\right)$ (Scoffoni et al. 2017). Importantly, when applied to a transpiring leaf, the SPC provides a capacitance-weighted average over the gradient in potential in the leaf tissue (as depicted by $\psi_{\mathrm{SPC}\;}$ in Fig. 1A) (Cochard et al. 2013). This averaging may obscure a substantial component of the water potential gradient within the OXZ, potentially leading to an overestimation of $K_{\mathrm{oxz}}$, incomplete assessments of the dependence of $K_{\mathrm{oxz}}$ on anatomy and water status (Buckley et al. 2015), and of the coupling of OXZ status to stomatal closure (Buckley and Mott 2013), embolism avoidance (Hochberg et al. 2017), and collapse of the leaf xylem conduits (Zhang et al. 2016). Other recent studies using isolated protoplasts (Shatil-Cohen et al. 2011; Sade et al. 2014), inference from gas exchange analysis (Wong et al. 2022), and isotope discrimination (Cernusak et al. 2018; Holloway-Phillips et al. 2019) have suggested that the dynamic regulation of hydraulic conductance of the plasma membrane could decrease $K_{\mathrm{oxz}\;}$ to levels comparable to that of stomata. An extreme loss of the conductance along the transpiration pathway might explain observations of substantial undersaturation (<85%RH) at the sites of evaporation (Rockwell et al. 2022). If leaves can indeed sustain such an unsaturated state in their airspaces, it would challenge the current consensus on leaf water transport that stomata are uniquely effective at regulating water loss from leaves (Buckley 2019; Rockwell et al. 2022).

Further progress has been hindered by the lack of practical, in situ, nondestructive methods to resolve water potentials and conductance in the OXZ. In this paper, in leaves of maize (*Zea mays* L.), we use a nanogel reporter of water potential (AquaDust (AQD)—Fig. 1B—(Jain et al. 2021)) in combination with gas exchange (Fig. 1C) and selective blocking of transpiration from leaf surfaces to access potentials upstream (at xylem, $\psi^{\mathrm{xyl}}$), inside (at bundle-sheath mesophyll/air interface, $\psi^{\mathrm{bs}}$), and downstream (in terminal evaporating surfaces of mesophyll adjacent to stomata, $\psi^{\mathrm{term}}$) of the OXZ (Fig. 1D). With these localized potentials, we extract values for the conductances of the leaf $\left(K_{\mathrm{leaf}}\right)$, the whole OXZ $\left(K_{\mathrm{oxz}}\right)$, the bundle sheath $\left(K_{\mathrm{bs}}\right)$, and the abaxial mesophyll $\left(K_{m}^{\mathrm{ab}}\right)$ and adaxial mesophyll ($K_{m}^{\mathrm{ad}}$, Fig. 1B) as a function of water status ($\psi^{\mathrm{xyl}}$, see Table 1 for definitions for acronyms). These measurements and manipulations provide a basis for the dissection of the water relations of the OXZ and open an unprecedented view of the individual contributions of the xylem, bundle sheath, and mesophyll to whole-leaf conductance and its regulation.

## Results

### Accessing localized values of water potential with AquaDust reporter

AquaDust is a nanoscale gel in which the Förster resonance energy transfer (FRET) between two covalently linked dyes provides calibrated changes in the fluorescence spectra as a function of changes in water potential in the gel's local environment (see Supplementary Methods S1 and Jain et al. (2021) for details). Upon infiltration through stomata into the interior of a leaf (see Supplementary Methods S2 for details), AquaDust distributes itself throughout the mesophyll, coating the surfaces of the intracellular air spaces; it does not enter the symplast or the xylem (red trace in Fig. 1A; Materials and Methods and Supplementary Movie S1). This mode of deposition provides a form of localization: the nanoreporters sit at the interface between the condensed phase of water in cell walls and the vapor phase that together make up the apoplast of the mesophyll.

**Figure 2.**
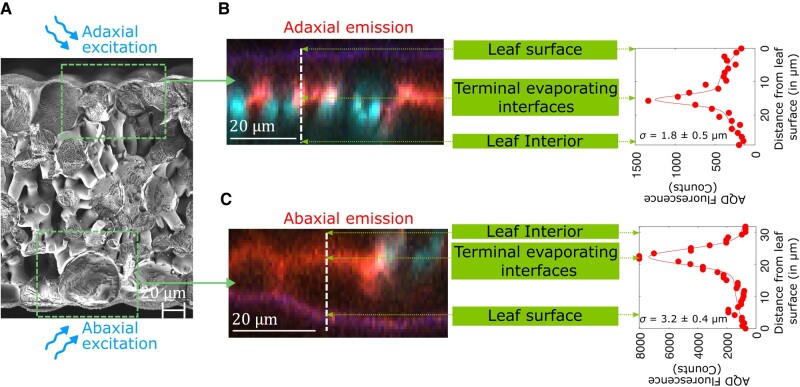
Localization of AquaDust optical signal. **A) to C)** Cryo-SEM micrograph of a **A)** maize leaf cross-section and the depth profile of acceptor dye emission (580 ± 10 nm) from fluorescence confocal cross-sections through **B)** adaxial and **C)** abaxial surfaces of maize leaf. Native autofluorescence from cytosol, cuticle, and chloroplast is false-colored as blue and AQD fluorescence is false-colored as red. See Supplementary Movie S1 for top-view, *z*-stack confocal micrographs.

A second form of localization comes from the optics of the fluorescent measurement: the fluorescence signal captured from the particles is localized within 15−25μm of the inner epidermal surface of the side, adaxial, or abaxial, from which the measurement is performed (as labeled in Figs. 1A and 2). We interpret this localization as being due to optical absorption and scattering of both the excitation and emission light interacting with different components of the leaf tissue made up of different refractive indices. Based on these two forms of localization, we hypothesize that AquaDust measurements provide access to a local average of the water potential at the terminal sites of evaporation adjacent to stomata ($\psi_{\mathrm{AQD}}≅\psi^{\mathrm{term}}$, Fig. 1, A and D(ii)), the extreme end of the hydraulic path followed by transpiration from soil to the atmosphere (see Materials and Methods and Supplementary Methods S3 for an additional explanation of methods and theory).

**Figure 3.**
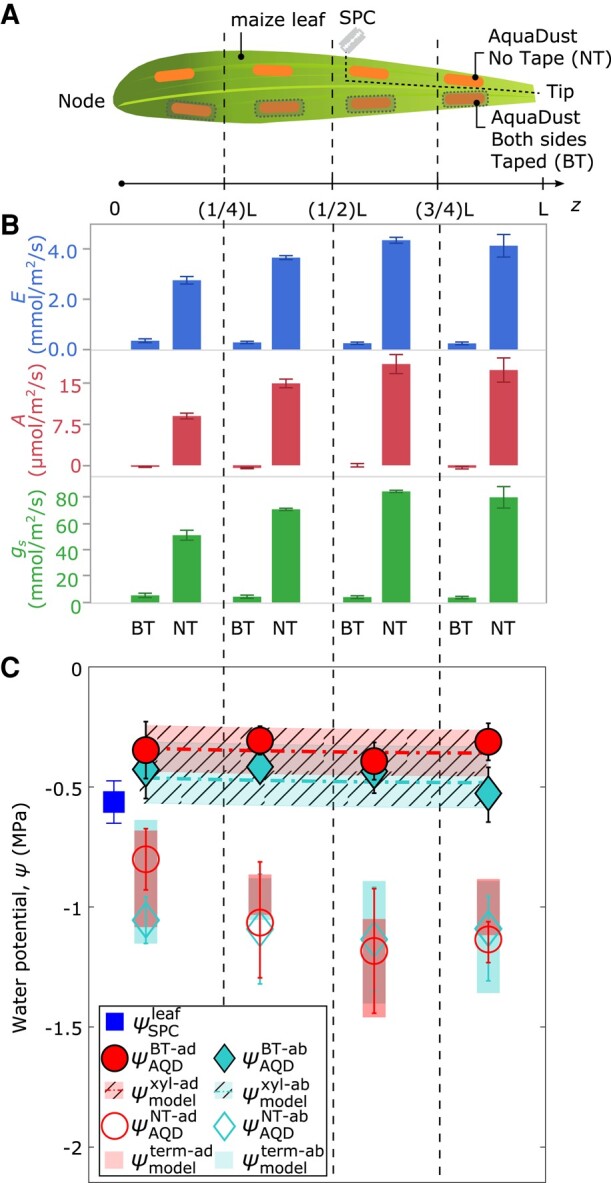
AquaDust measurements with controlled transpiration along the length of the maize leaf. **A)** Diagram of maize leaf with eight regions infiltrated with AquaDust (orange shaded) and either without any tape on either side (NT) or covered on both sides with impermeable tape (BT). One SPC measurement was performed on a whole leaf (cut at the indicated position) at the end of experiment. At the end of the experiment, one SPC measurement was taken on the leaf tissue. This tissue was excised by cutting along the dashed lines labeled with a diagram of a razor blade (see Materials and Methods for details). **B)** Transpiration rate (E, mmol/$m^{2}/s)$, assimilation rate (A, $\mu \mathrm{mol}/m^{2}/s$), and stomatal conductance $(g_{s},\mathrm{mmol}/m^{2}/s$) in each zone on BT or NT regions (n=4, error bars represent standard error). **C)** Potentials measured with AquaDust (*n* = 4, error bars represent standard error) on the adaxial side for NT and BT regions ($\psi_{\mathrm{AQD}}^{\mathrm{NT}-\mathrm{ad}}$ and $\;\psi_{\mathrm{AQD}}^{\mathrm{BT}-\mathrm{ad}}$) and abaxial side for NT and BT regions ($\psi_{\mathrm{AQD}}^{\mathrm{NT}-\mathrm{ab}}$ and $\psi_{\mathrm{AQD}}^{\mathrm{BT}-\mathrm{ab}}$) compared to model predictions of xylem water potential ($\psi_{\mathrm{model}}^{\mathrm{xyl}}$—shaded) and terminal water potential ($\psi_{\mathrm{model}\;}^{\mathrm{term}\;}$—unshaded). Model predictions are based on a hydraulic resistance framework that partitions resistance between xylem and outside xylem tissues. Refer to Supplementary Fig. S1, and Jain et al. (2021) for model details.

To access values of water potential at other locations in the cross-section of the leaf, we selectively block the transpiration from the areas of the leaf surface from which we capture the AquaDust signal (Fig. 1D). Figure 3A presents this strategy on four segments along the length of a maize leaf: in each segment, we infiltrated AquaDust into one region on each side of the midrib; we leave four of these regions uncovered (orange regions along top half of leaf—no taped (NT)) and cover both the adaxial and abaxial surfaces of the other four regions with a transparent tape to obstruct transpiration (shaded orange regions on the bottom half of leaf—both taped (BT)—See Materials and Methods and Supplementary Fig. S1 for details). The measurements of transpiration rate (E), assimilation rate (A), and effective stomatal conductance $\left(g_{s}\right)$ captured from these regions demonstrate the efficacy of the tape for suppressing gas exchange (Fig. 3B); all values dropped by >90% relative to the adjacent uncovered regions. With transpiration obstructed on both sides of the leaf (BT), we assume equilibrium through the full thickness of the leaf (Fig. 1D(i)) such that $\psi_{\mathrm{AQD}}^{\mathrm{BT}-\mathrm{ad}}≅\psi_{\mathrm{AQD}}^{\mathrm{BT}-\mathrm{ab}}≅\psi^{\mathrm{xyl}}$, where $\psi^{\mathrm{xyl}}$ is the local potential in the xylem. In Fig. 3C, we plot the potentials reported by AquaDust in these BT regions (closed symbols); we observe agreement within uncertainty between adaxial $\left(\psi_{\mathrm{AQD}}^{\mathrm{BT}-\mathrm{ad}}\right)$ and abaxial $\left(\psi_{\mathrm{AQD}}^{\mathrm{BT}-\mathrm{ab}}\right)$ values with model predicted values of local xylem water potential $\left(\psi_{\mathrm{model}}^{\mathrm{xyl}}\right)$ along the length of the leaf using the xylem conductance in maize based on destructive sampling (Li et al. 2009) (dashed curves—Supplementary Fig. S1, details of the model are described by Jain et al. (2021)). These methods provide an unprecedented basis for capturing local xylem potential, upstream of the OXZ, on intact leaves.

**Figure 4.**
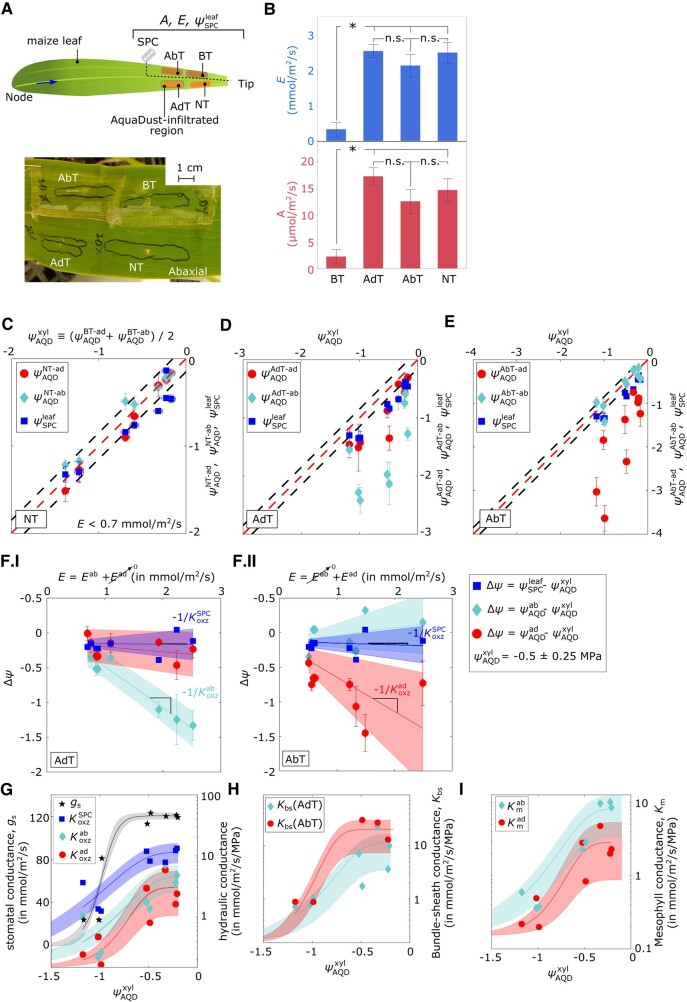
Interrogation of local leaf hydraulics with AquaDust in maize. **A)** Schematic diagram and picture of a maize leaf with four different treatments to manipulate adaxial and abaxial transpirations $(E^{\mathrm{ad}}$ and $E^{\mathrm{ab}})$: suppressing transpiration (E) by applying tape on both sides (BT), applying tape on AdT, applying tape on AbT, and a control region with NT (see Fig. 1D). Dashed lines labeled with a diagram of a razor blade show the section of the leaf tissue section that was cut and used to measure leaf water potential with a Scholander pressure chamber $(\psi_{\mathrm{SPC}}^{\mathrm{leaf}}$). Arrows indicate the approximate length of the section of leaf tissue used for leaf water potential measurement using Scholander pressure chamber $\left(\psi_{\mathrm{SPC}}^{\mathrm{leaf}\;}\right)$. **B)** Transpiration rate $\left(E,\mathrm{mmol}/m^{2}/s\right)$, and assimilation rate $\left(A,\mu \mathrm{mol}/m^{2}/s\right)$ with four cases (BT, AdT, AbT, and NT) that correspond to the sites of measurements in **A)** (n=4, error bars represent the standard error). Two-tailed t-test confirms significant decline (*P* < 0.05) in *E* and *A* from the BT region compared to the AdT, AbT, and NT regions (*: *P*-value < 0.05, n.s.: not significant, see Supplementary Table S1 for quantitative values and analysis). See Materials and Methods (Eqs. 1 to 8) for the calculation of the various hydraulic conductances from these potentials. Conductances and architecture in each treatment area are as in Fig. 1A. **C) to E)** Measured values of whole-leaf potential ($\psi_{\mathrm{SPC}}^{\mathrm{leaf}\;}$—squares) and AQD potentials at adaxial $\psi_{\mathrm{AQD}}^{\mathrm{ad}}$—circles) and abaxial $\psi_{\mathrm{AQD}}^{\mathrm{ab}}$—diamonds) sides of the leaf (sample size: 14 plants, each point corresponds to one plant; error bars represent the standard error for 3 to 6 measurements collected per plant; see Materials and Methods and Supplementary Methods S3 for more details) as a function of xylem potential $\left(\psi_{\mathrm{AQD}}^{\mathrm{xyl}}\right)$ for dark-adapted leaves with low transpiration rate ($E<0.7\;\mathrm{mmol}/m^{2}/s$—**C**), for transpiring leaves $\left(E^{\mathrm{ab}}>0.7\;\mathrm{mmol}/m^{2}/s\right)$ with adaxial side taped (AdT—**D**), and for transpiring leaves $(E^{\mathrm{ad}\;}>0.7\;\mathrm{mmol}/m^{2}/s$) with abaxial side taped (AbT—**E**). Red and dashed lines are one-to-one; black and dashed lines show uncertainty in the calibration of AquaDust (±0.15MPa (Jain et al. 2021)). **F)** Deviation (Δψ) of $\psi_{\mathrm{SPC}}^{\mathrm{leaf}\;}$ (squares), $\psi_{\mathrm{AQD}}^{\mathrm{ab}}$ (diamonds), and $\psi_{\mathrm{AQD}}^{\mathrm{ad}}$ (circles) from $\psi_{\mathrm{AQD}}^{\mathrm{xyl}}$ as a function of **F.I)**$E^{\mathrm{ab}}$ for the case of AdT zone and **F.II)**$E^{\mathrm{ad}}$ for the case of AbT zone in well-watered maize $\left(\psi_{\mathrm{AQD}}^{\mathrm{xyl}}=-0.5\pm 0.25\;\mathrm{MPa}\right)$ (sample size of 7 plants; each point corresponds to one plant; error bars represent standard error). Lines represent linear regression fit, and shaded zones represent 95% confidence intervals (see Supplementary Tables S2 and S3 for details). **G)** Variation of stomatal conductance ($g_{s}$—black stars), effective leaf conductance ($K_{\mathrm{oxz}\;}^{\mathrm{SPC}\;}$ squares—Eq. 1), adaxial and abaxial outside xylem conductance ($K_{\mathrm{oxz}}^{\mathrm{ad}}$ and $K_{\mathrm{oxz}}^{\mathrm{ab}}$—circles and diamonds—Eqs. 3 and 4) as a function of xylem potential based on AquaDust measurement in BT region $\left(\psi_{\mathrm{AQD}}^{\mathrm{xyl}}\right)$. **H) and I)** Variation of bundle-sheath conductance $K_{\mathrm{bs}}$ (**H**) and of $K_{m}^{\mathrm{ad}}$ and $K_{m}^{\mathrm{ab}}$ (**I**), calculated for the AdT and AbT zones as a function of $\psi_{\mathrm{AQD}}^{\mathrm{xyl}}$ (see Materials and Methods Eqs. 1 to 8, see Supplementary Table S4, S5, and S6 for definitions of effective conductances and fits (sigmoidal curves with shaded confidence intervals)).

The open symbols in Fig. 3C present the potentials reported by AQD from the adjacent, unobstructed regions (NT) in each segment of the leaf. As indicated schematically in Fig. 1D(ii), we hypothesize that these values represent the terminal potentials at the downstream end of the OXZ during active transpiration: $\psi_{\mathrm{AQD}}^{\mathrm{NT}-\mathrm{ad}}≅\psi^{\mathrm{term}\text{-}\mathrm{ad}\;}$ and $\psi_{\mathrm{AQD}}^{\mathrm{NT}-\mathrm{ab}}≅\psi^{\mathrm{term}\text{-}\mathrm{ab}\;}$. The observed values (open symbol—Fig. 3C) are consistent with this interpretation (Supplementary Fig. S1): they fall significantly below both the values in the xylem (closed symbols—Fig. 3C) and the potential of the whole leaf captured with the SPC (blue square—Fig. 3C). Model predictions (shaded bars in Fig. 3C) based on our detailed interrogation of the variable conductance of the OXZ (Fig. 4) agree well with these measurements of $\psi^{\mathrm{term}\;}$. Local access to the potentials both upstream (i.e. in BT scenarios) and downstream (i.e. in NT scenarios) of the OXZ provides a basis for assessing the effects of transpiration on the state of water in leaf mesophyll tissue, the dominant locus of terrestrial photosynthesis.

### Hyper-local water relations of the OXZ in maize in response to stress

In Fig. 4, we present a deeper interrogation of the water transport in the distal segment of maize leaves (Fig. 4A). When one side of a leaf is blocked while the opposite surface is transpiring, we expect that the potential of the blocked surface should be close to that of the bundle sheath surface, an expectation motivated by the prediction for distribution of *ψ* through the leaf cross-section using finite-element models of water transport in leaves (see Materials and Methods, Supplementary Figs. S2 and S3, (Rockwell et al. 2014a, 2014b) for a detailed description of definitions, methods, and hydraulic architecture). To exploit this idea, we infiltrated four regions with AQD (Fig. 4A) and subjected them to four different taping treatments: (i) blocked transpiration at both surfaces (BT); (ii) unobstructed transpiration (NT); (iii) blocked transpiration at the adaxial surface only (AdT); and (iv) blocked transpiration at the abaxial surface only (AbT). In Fig. 4B, we reported the assimilation rate and transpiration rate from these regions (see Supplementary Table S1 for details). We followed the response of these regions to increasing xylem stress (induced by soil drying), from near saturation to the turgor loss point ($-1.5<\psi^{\mathrm{xyl}}<-0.2\mathrm{MPa}$, Fig. 4C to E).

As above, we interpret the doubly taped potentials as indicative of the state of the local xylem in the BT region $\left(\psi_{\mathrm{AQD}}^{\mathrm{xyl}}=(\psi_{\mathrm{AQD}}^{\mathrm{BT}-\mathrm{ad}}+\psi_{\mathrm{AQD}}^{\mathrm{BT}-\mathrm{ab}})/2\right)$. At minimal levels of transpiration (in dark, $E<0.7\mathrm{mmol}/m^{2}/s$), we see the convergence of the values in the NT region: $\psi^{\mathrm{xyl}}≅\psi_{\mathrm{SPC}}^{\mathrm{leaf}}≅\psi_{\mathrm{AQD}}^{\mathrm{ad}}≅\psi_{\mathrm{AQD}}^{\mathrm{ab}}$ (Fig. 4C; black dashed lines represent the uncertainty of AQD measurements (±0.15MPa) (Jain et al. 2021)). This convergence is consistent with our expectation of near equilibrium of all components of the leaf at low transpiration fluxes, and thereby provides an in-planta validation of the accuracy of AquaDust relative to the SPC.

At higher rates of transpiration $\left(E>0.7\mathrm{mmol}/m^{2}/s\right)$ with blocked adaxial (AdT—Fig. 4D) and blocked abaxial (AbT—Fig. 4E) surfaces, the water potentials at the transpiring surfaces deviate strongly from the xylem (red dashed, one-to-one line) as expected, but also from the SPC ($\psi_{\mathrm{SPC}}^{\mathrm{leaf}\;}$—blue squares), supporting our hypothesis that the localization of AquaDust captures a transpiration induced gradient of potential in the OXZ that is inaccessible to the SPC. Further support for this idea comes from leaves transpiring at varying rates at low xylem stress: the pressure gradient defined by AquaDust responds linearly to transpiration, implying a constant conductance of the OXZ in unstressed leaves, while the gradient defined by the SPC is unresponsive to transpiration (Fig. 4F.I, and II, see Supplementary Tables S2 and S3 for quantitative estimates). Finally, we observe in some cases, particularly, with declining $\psi_{\mathrm{AQD}}^{\mathrm{xyl}}$ and $\psi_{\mathrm{SPC}}^{\mathrm{leaf}}$, that terminal $\psi^{\mathrm{oxz}}$ is substantially more negative than the average turgor loss point for these species as defined by the SPC, even though these leaves continue to have reasonable transpiration and assimilation rates, without signs of macroscopic loss of turgor (Fig. 4A).

Our localized measurements can also be used to capture the response of the OXZ to xylem stress. Using the water potential data reported in Fig. 4, D and E for maize leaves at increasing levels of stress down to turgor loss, we provide direct assessments of a decline in the conductance of the OXZ paths to the abaxial ($K_{\mathrm{oxz}}^{\mathrm{ab}}$, cyan diamonds) and adaxial ($K_{\mathrm{oxz}}^{\mathrm{ad}}$, red circles) surfaces (Fig. 4G). We make the following observations from these measurements: (i) we note that substantial symmetry between the OXZ conductances to the adaxial and abaxial surfaces, with overlapping prediction intervals (shaded cyan and pink zones) for the best-fit sigmoidal responses (solid red and cyan curves) and similar values of potential at 50% loss of conductance ($\psi_{50\text{\%}}≅-0.6\;\mathrm{MPa}$, Supplementary Table S4); this symmetry is consistent with that of the morphology of the isobilateral leaves of maize (Fig. 2A); (ii) we confirm that conventional measurements based on the SPC (blue squares—Fig. 4G) underestimate gradients in the OXZ: $K_{\mathrm{oxz}}^{\mathrm{SPC}}$ as defined by using the difference in bulk tissue water potential and local measurement of local xylem water potential (see Materials and Methods, Eqs. 1 and 2 for details for definition of conductances) is 2- to 10-fold above that assessed with AquaDust and shows only a 3-fold drop over the range of stress studied compared to the 10-fold drop in conductance assessed using AquaDust; (iii) we see in the overlaid trend in $g_{s}$ (black stars) that the OXZ conductance drops earlier (at higher potentials) and further than the stomatal conductance (see Supplementary Table S4 for quantitative values of fit parameters and Supplementary Fig. S4 for plots of resistances); (iv) finally, we note that we can use the best-fit curves for $K_{\mathrm{oxz}}^{\mathrm{ad}}$ and $K_{\mathrm{oxz}}^{\mathrm{ab}}$ to model the variations in potential observed along the length of whole leaves (Fig. 3C—unshaded translucent red and cyan; see Supplementary Fig. S1 for details).

The weak but resolvable deviations of potentials to the blocked surfaces opposite transpiring surfaces support our hypothesis that AquaDust signals from a non or minimally-transpiring side of a leaf can provide access to the water potential at the bundle sheath to mesophyll transition (Fig. 1A, $\psi_{\mathrm{AQD}}^{\mathrm{AdT}-\mathrm{ad}}$—red circles in Fig. 4D; $\psi_{\mathrm{AQD}}^{\mathrm{AbT}-\mathrm{ab}}$—cyan diamonds in Fig. 4E; see Materials Methods and Supplementary Fig. S2 for details). No existing technique provides access to these contributions in situ, yet indirect techniques (e.g. permeability measurements on protoplasts) suggest distinct regulation of water conductance by the bundle sheath and mesophyll (Shatil-Cohen et al. 2011) and molecular mechanisms for this regulation (Grunwald et al. 2022). This technique allows us to construct separate variability curves for the conductances of the bundle sheath $\left(K_{\mathrm{bs}}\right)$ and mesophyll $\left(K_{m}\right)$ as shown in Fig. 4, H and I (see Materials and Methods Eqs. 1 to 8 for the definition of conductances, Supplementary Tables S5 and S6 for values of fit parameters, and Supplementary Fig. S4B, and C for plots of resistances). We note here that we only include the data from Fig. 4, D and E in Fig. 4H where the deviation from $\psi_{\mathrm{AQD}}^{\mathrm{xyl}}$ is resolvable (>±0.15MPa) using AquaDust. With this deeper dissection of the water conductance in the OXZ, we find that both the bundle sheath and mesophyll tissues contribute to the loss of conductance with similar values of $\psi_{50\text{\%}}≅0.6\;\mathrm{MPa}$, suggesting that these tissues may share both the mechanism of regulation and the functional roles associated with lowering of conductance in response to water stress.

## Discussion

### Interpretation and implications of terminal potentials in mesophyll and variation in OXZ conductance

AquaDust provides measurements of water potentials at the boundaries of the OXZ: the source end is defined by the local xylem water potential $\left(\psi^{\mathrm{xyl}}\right)$ at a given location along the length of the leaf, and the sink end is defined by the water potential of the terminal evaporating surfaces ($\psi^{\mathrm{term}}$) underneath the epidermis. We have investigated the changes in the tissue conductance $\left(K_{\mathrm{oxz}}\right)$ that couple the source and sink potentials with independent control of the environmentally relevant variables, i.e. soil water supply that defines water availability to the plant and vapor pressure deficit (VPD) that defines the evaporative demand (E) from the plant. Access to the $\psi^{\mathrm{xyl}},\;\;\psi^{\mathrm{term}}$, and *E* with varying water availability and evaporative demand help resolve important and persistent uncertainties in our understanding of the water relations of the OXZ relative to hydraulic architecture, constitutive properties, and the sites of dominant and variable conductances (Buckley 2015; Buckley et al. 2015; Rockwell and Holbrook 2017; Scoffoni et al. 2017).

We found that the passage of water through the OXZ creates a substantial drop in *ψ*; Δψ across OXZ is of the same order or greater than that across all upstream components of the soil-plant-atmosphere continuum. Using AquaDust to access the potential at the sites of evaporation, we find that the conductance of the OXZ is ∼200% lower than previously measured using other techniques, with low values of $K_{\mathrm{oxz}\;}$ being $∼0.2-0.3\;\mathrm{mmol}/m^{2}/\mathrm{MPa}/s$ as measured here compared to $∼3-5\mathrm{mmol}/m^{2}/\mathrm{MPa}/s$ measured using pressure-probe method (Shackel and Brinckmann 1985; Shackel 1987) or $∼1\;\mathrm{mmol}/m^{2}/\mathrm{MPa}/s$ (Trifiló et al. 2016; Scoffoni et al. 2017) measured using SPC in cases when $\psi^{\mathrm{xyl}}>\psi^{\mathrm{TLP}}$. Notably, we observe in some cases, particularly with declining $\psi^{\mathrm{xyl}}$, that $\psi^{\mathrm{term}\;}$ became substantially more negative than the turgor loss point of maize, even as these leaves continued to have reasonable transpiration and assimilation rates and show no signs of macroscopic loss of turgor. While surprising, this observation aligns with recent assessments of *ψ* using indirect techniques, where substantial unsaturation $\left(\psi^{\mathrm{term}\;}\ll \psi^{\mathrm{TLP}}\right)$ was reported in leaves of oneseed juniper (*Juniperus monosperma*), pine (*Pinus edulis)*, cotton (*Gossypium hirsutum* L.), and sunflower (*Helianthus annuus* L.) (Cernusak et al. 2018; Wong et al. 2022). Since AquaDust is localized in the apoplast, the observation that $\psi^{\mathrm{term}\;}<\psi^{\mathrm{TLP}\;}$ suggests two distinct possibilities: (i) locally, cells proximate to stomata lose turgor, or, (ii) there is substantial resistance at the interface between the symplast and the apoplast resulting in the breakdown of local equilibrium between water in liquid and vapor phase. Either of these possibilities has wide-ranging implications in our understanding of water transport in OXZ and requires further investigation (Rockwell et al. 2022; Wong et al. 2022).

Our direct measurements of $K_{\mathrm{oxz}}$ in physiologically relevant conditions of intact, actively assimilating and transpiring, leaves allow examination of two hypothetical scenarios explored in the literature for mechanism and functional importance of the decline in $K_{\mathrm{oxz}}$ (Shatil-Cohen et al. 2011; Trifiló et al. 2016; Scoffoni et al. 2017; Scoffoni and Sack 2017; Wong et al. 2022). In the first, the decline in $K_{\mathrm{oxz}}$ is associated with passive, physical processes resulting from the dehydration of the OXZ tissue and morphological changes such as shrinkage and drying (with capillary failure) of cell walls (Trifiló et al. 2016; Scoffoni et al. 2017; Scoffoni and Sack 2017); we refer to this scenario as apoplastic vulnerability, in analogy to the physical loss of conductance observed in xylem at extreme stress (typically beyond the turgor loss point). In the second scenario, the decline in $K_{\mathrm{oxz}}$ is associated with regulation of the conductance to the transpiration stream within the bundle sheath and mesophyll, likely via cell biologically controlled changes in the permeability of plasma membranes; we refer to this scenario as active regulation (Shatil-Cohen et al. 2011; Scoffoni et al. 2017; Wong et al. 2022), to emphasize its potential mechanistic and functional connections to the active processes associated with stomatal regulation.

Studies of tissue-specific contributions involving measurements of membrane permeability of protoplast (Sade et al. 2014; Grunwald et al. 2022) have linked both bundle sheath and mesophyll tissue to the decline in $K_{\mathrm{oxz}\;}$. Our measurements confirm that both these tissues (Fig. 4, H and I) contribute to the overall decline in $K_{\mathrm{oxz}}$. Yet, the mechanisms driving declines in $K_{\mathrm{oxz}}$ remain controversial. A leading hypothesis proposes that, due to the presence of an apoplastic barrier, bundle-sheath conductance depends upon biologically regulated plasma membranes, while mesophyll conductance is dominated by an apoplastic path that contributes to declines in $K_{\mathrm{oxz}\;}$ by passive dehydration (Taneda et al. 2016; Buckley et al. 2017; Scoffoni and Sack 2017). Against this latter idea, we note that the loss of hydraulic connection due to capillary failure within the cell walls at the potentials observed in $\psi^{\mathrm{term}\;}$ would also require a large pore size (∼100nm), an order of magnitude larger than the pore size typically observed in cell wall matrix (Dietz and Herth 2011). Furthermore, we observe that the OXZ conductance remains relatively constant as a function of *E* (constant slope—Fig. 4F), implying that $K_{\mathrm{oxz}}$ is only a weak function of evaporative demand. This observation is inconsistent with the hypothesis that the decline in $K_{\mathrm{oxz}}$ results from the passive loss of conductance in the cell wall since this process should be more sensitive to increasing evaporative demand than xylem water potential.

Rather than being highly sensitive to changes in evaporative demand (VPD) and the resulting transpiration rate (Fig. 4F), we find $K_{\mathrm{oxz}}$ to be a strong function of $\psi^{\mathrm{xyl}\;}$ (Fig. 4G). This decline in $K_{\mathrm{oxz}\;}$ takes place at a higher value of xylem water potential, before the water stress that leads to either stomatal closure or turgor loss, favoring the hypothesis that the decrease in OXZ conductance is actively regulated within the photosynthetically active OXZ tissue to protect xylem from large tensions while keeping the stomata open for carbon uptake (Wong et al. 2022). We note that the concomitant decline in bundle-sheath and mesophyll conductance with similar values of $\psi_{50\text{\%}}(≅0.6\mathrm{MPa})$ suggests a shared regulatory mechanism for water transport in both bundle-sheath and mesophyll cells. Finally, we observed that the total conductance of the OXZ falls substantially further than that of the stomata and at higher values of xylem water potential. Taken together, our measurements suggest that this active regulation of the OXZ may be an adaptive response that protects upstream components of the transpiration path and plays a role in modulating gas exchange.

## Conclusion

The local interrogation of OXZ achieved with AquaDust reveals large gradients of water potential in the outside xylem tissue and dramatic loss of conductance of both bundle-sheath and mesophyll tissue in response to drought stress in the xylem. Our observations are consistent with the active regulation of OXZ conductance by cellular responses triggered by the declining water availability encoded in the water potential in the xylem. Our observation of apoplastic potentials well below the turgor loss point brings into question the long-standing assumption of local equilibrium between apoplast and symplast of the mesophyll tissue and the related assumption that the tissue in the OXZ may be safely represented as homogeneous continuum tissue-level properties. The large decline in the conductance of OXZ has implications for coupled processes of water loss and CO_2_ assimilation that are hosted by OXZ; this tissue may provide a selective control that can limit transpiration without compromising the assimilation of CO_2_. This study presents a set of tools for further interrogation of these OXZ phenomena in the contexts of crop improvement, ecophysiology, and land-atmosphere modeling in a changing climate.

## Materials and methods

### Plant material and growing conditions

#### Species selection and properties

Maize (*Zea mays* L., Inbred line B97) leaves present an isobilateral leaf anatomy. Stomatal distribution and density were calculated from micrographs of leaves collected using confocal microscope images (see details in the next subsection). The stomatal distribution in “B97” maize variety used in this report has 41% of the total stomata $\left(69\pm 5\mathrm{stomata}/mm^{2}\right)$ on the adaxial and 59% of the total stomata $\left(98\pm 6\mathrm{stomata}/mm^{2}\right)$ on the abaxial side of the leaf respectively.

#### Growing conditions

Maize plants were grown from seed in a 1:1 ratio of Cornell Mix (Landry et al. 1995) and Turface (Turface Athletics MVP Profile, Products, LLC; Buffalo Grove, IL, USA) in 10-cm diameter, 8.4-cm deep nursery pots (volume of 400ml). Plant material was grown under controlled conditions in a walk-in growth chamber with a 10-hour photoperiod, $22^{\circ }C$ day and $20^{\circ }C$ night temperature, 50% relative humidity, and a photosynthetic photon flux density of $270\mu \mathrm{mol}/m^{2}/s$ PPFD. Plants were kept well-watered during the growth period. Maize plants were fertilized twice a week with Jack's 21-5-20 Fertilizer (JR Peters Inc, Allentown, PA, USA) at 200 ppm. Experiments were conducted when maize plants were between 6 and 8 weeks (growth stage V5–V7).

### AquaDust and its use for measurements of water potential in maize

#### Synthesis

AquaDust nanoparticles (30 to 100 nm (range) diameter) were formed of hydrogel containing two distinct fluorescence dyes covalently linked to the matrix (Fig. 1B) as described previously by Jain et al. (2021) and in Supplementary Methods S1.

#### Use of AquaDust in maize (Figs. 3 and 4)

Plants were infiltrated with a suspension of AquaDust with a syringe as described in Supplementary Methods S2, and measurements were taken starting 24 h later, after excess water from the injection had dissipated. The AquaDust spectra were collected from the adaxial and abaxial sides of the leaf in areas that had been infiltrated with the suspension using a commercial reflection probe (QR600-7-UV-125F Premium 600-micron Reflection Probe, Ocean Optics Inc.) and spectrometer (ST2000, Ocean Optics Inc.), as described in Supplementary Methods S3. These spectra were analyzed and calibrated, as described previously by Jain et al. (2021) and in Supplementary Methods S3. At the end of experiments on a leaf with AquaDust, leaf water potentials $\left(\psi_{\mathrm{leaf}\;}^{\mathrm{SPC}}\right)$ of the AquaDust injected leaf and of an adjacent leaf were taken by cutting along the dashed lines in Figs. 3A and 4A with a razor blade and immediately inserting it into a Scholander pressure chamber to determine the balancing pressure (the pressure at which a water droplet became visible on the cut surface).

#### Localization of AquaDust signal in the mesophyll (Fig. 2)

We obtained the depth profiles (Fig. 2, B and C) of fluorescence intensity of acceptor dye emission (580±10nm) on the adaxial and abaxial leaf sides of maize leaves with a laser scanning confocal microscope (Zeiss LSM880). An intact maize leaf infiltrated with AquaDust was mounted on a glass slide using a double-sided tape, placed on Zeiss LSM880 Confocal Upright Microscope, and imaged using W Plan-Apochromat 20×/1.0 water immersion objective and a 561nm excitation laser line. These depth profiles were obtained by constructing orthogonal sections and plotting intensity as a function of depth in these orthogonal sections using ZEN software and MATLAB. See Supplementary Methods S4 for details on sample preparation for cryogenic scanning electron micrographs in Fig. 2A.

#### Gas exchange measurements (Figs. 3 and 4)

Rates of transpiration (E), assimilation (A), and stomatal conductance $\left(g_{s}\right)$ were collected from regions infiltrated with AquaDust using a CIRAS-3 system (PPSystems, Amesbury, MA, USA). Measurements were taken after gas exchange parameters stabilized (typically in 2 to 3 min). Cuvette parameters were 400ppm of $CO_{2},$ 300 cc min-1 gas flow rate, and temperature, light levels, and VPD were set to match the ambient conditions in the growth chamber as described earlier in the Materials and Methods section on growing conditions, or as indicated in captions. The AquaDust spectrum was collected immediately after the gas exchange was performed on the corresponding region.

#### Measurement of xylem and outside xylem potentials along the length of leaves under well-water conditions (Fig. 3)

Four well-watered maize plants (biological replicates) were kept and grown in the growth chamber with environmental conditions as described in the previous subsection. These plants exhibited an average $\psi_{\mathrm{SPC}}^{\mathrm{leaf}\;}$ of ∼−0.55MPa (Fig. 3C—blue square). From each plant, one leaf, specifically leaf 6/7, was chosen for infiltration of eight regions with AquaDust (orange) as shown in Fig. 3A. These regions were evenly distributed on either side of the midrib within four zones along the leaf's length. In each of these zones, one AquaDust region was covered with non-fluorescent tape on both adaxial and abaxial sides (BT), while the other remained uncovered (NT). Figure 3B presents the data on transpiration rate (E), assimilation rate (A), and effective stomatal conductance $\left(g_{s}\right)$ obtained through gas exchange measurements on NT regions and BT regions (Buck 1981; von Caemmerer and Farquhar 1981).

#### Measurement of local xylem and outside xylem potentials through dry down (Fig. 4)

Fourteen leaves (leaf# 6/7) on 14 well-watered plants (biological replicates) were infiltrated with AquaDust on day 0 at four locations as shown in Fig. 4A. These regions were subjected to the following treatments using an optically transparent tape that is impermeable to gas exchange (low fluorescence optical transparent tape—Nunc Sealing, Thermo Scientific Inc., Waltham, MA, USA): (i) BT—taping both sides of the leaf such that $E^{\mathrm{ab}}+E^{\mathrm{ad}}≃0$, and (ii) NT—no taping, (iii) AdT—taping adaxial side of the leaf with a tape such that $E^{\mathrm{ad}}≃0$, and (iv) AbT—taping adaxial side of the leaf with a tape such that $E^{\mathrm{ab}}≃0$ (Fig. 4A).

Measurements were performed during the subsequent three-day period, days 1 to 3, in a growth chamber maintained at the conditions reported above. Water was withheld starting on day 2. On day 1, the evaporative demand for the well-watered plants $\left(\psi_{\mathrm{AQD}}^{\mathrm{xyl}}=-0.5\pm 0.25\mathrm{MPa}\right)$ was adjusted in the cuvette locally by altering the VPD between 0.7kPa and 2kPa with an increment of ∼0.2kPa. This modification resulted in different transpiration rates, as shown in Fig. 4F. On days 2 to 3, gas exchange and AquaDust measurements were performed on all infiltrated regions as xylem potential $\left(\psi_{\mathrm{AQD}}^{\mathrm{xyl}}\right)$ decreased through the dry down (Fig. 4C to E, G to I). Lastly, leaf water potential $\left(\psi_{\mathrm{SPC}}^{\mathrm{leaf}\;}\right)$ of the AquaDust injected leaf and an adjacent leaf were measured by cutting them with a razor blade as indicated in Fig. 4A and immediately inserting it into a Scholander pressure chamber to determine the balancing pressure (the pressure at which a water droplet became visible on the cut petiole surface).

### Analysis of xylem and outside xylem water relations

#### Analysis of xylem and outside xylem water potentials along the length of leaves (Fig. 3)

In Fig. 3C, the potentials measured with AquaDust in regions with obstructed (BT) and unobstructed (NT) gas exchange were compared to predictions based on a hypothetical hydraulic circuit model of the leaf (Supplementary Fig. S1B, and C) that represents each of the four axial zones in which measurements were performed (Fig. 3A). In each segment, the xylem conductance $\left(K_{\mathrm{xyl}}\right)$ and outside xylem conductance $\left(K_{\mathrm{oxz}}\right)$ depend on the local values of water potential ($\psi^{\mathrm{xyl}},$$\psi^{\mathrm{term}\text{-}\mathrm{ad}\;}$, and $\psi^{\mathrm{term}\text{-}\mathrm{ab}\;}$ respectively).

The variable xylem conductance, $K_{\mathrm{xyl}}(\psi^{\mathrm{xyl}})$ was based on a previously reported vulnerability function and associated parameter values ((Chunfang et al. 1999; Li et al. 2009)—see Supplementary Fig. S1D for parameters). The variable outside xylem conductance, $K_{\mathrm{oxz}\;}(\psi )$ was based on the vulnerability curve generated in this study and reported in Fig. 4G (see Supplementary Table S4 for values of parameters). In the model, we used the range of values measured for $\psi_{\mathrm{AQD}}^{\mathrm{NT}-\mathrm{ad}}$ and $\psi_{\mathrm{AQD}}^{\mathrm{NT}-\mathrm{ab}}$ in Zone 1 as a boundary condition for $\psi_{\mathrm{model}\;}^{\mathrm{term}\text{-}\mathrm{ad}\;}$ and $\psi_{\mathrm{model}\;}^{\mathrm{term}\text{-}\mathrm{ab}\;}$ respectively at z=1/8. Note that this hydraulic model does not require values of the soil water potential. The predictions from the model for xylem water potential using the adaxial and abaxial vulnerability curves are reported as $\psi_{\mathrm{model}\;}^{\mathrm{xyl}-\mathrm{ad}}$ and $\psi_{\mathrm{model}\;}^{\mathrm{xyl}-\mathrm{ab}}$ respectively in Fig. 3C.

#### Evaluation of outside xylem conductance and the contributions from the mesophyll and bundle sheath (Fig. 4)

The values of water potential and gas exchange rates reported in Fig. 4B to F were used to evaluate various conductances (Fig. 4G to I). First, conventional effective outside xylem conductance was calculated using Scholander pressure chamber for measuring water potential (Scoffoni et al. 2017):


(1) $$K_{\mathrm{oxz}}^{\mathrm{SPC}}=\frac{E^{\mathrm{ab}}+E^{\mathrm{ad}}}{\psi_{\mathrm{SPC}}^{\mathrm{leaf}}-\psi_{\mathrm{AQD}}^{\mathrm{xyl}}}$$

where xylem water potential was measured using AquaDust from BT region:


(2) $$\psi_{\mathrm{AQD}}^{\mathrm{xyl}}=\frac{\psi_{\mathrm{AQD}}^{\mathrm{BT}-\mathrm{ad}}+\psi_{\mathrm{AQD}}^{\mathrm{BT}-\mathrm{ab}}}{2}$$

Additionally, using AquaDust, gradients in *ψ* in the OXZ were accounted for the calculation of the total conductance of the OXZ tissue between the xylem and the sites of evaporation adjacent to the inner surfaces of the epidermis ($K_{\mathrm{oxz}}^{\mathrm{ab}}$ and $K_{\mathrm{oxz}}^{\mathrm{ad}}$). The values collected with the adaxial side taped (Figs. 1, A and D(ii), 4, B and D) were used to interrogate the abaxial side:


(3) $$\;\mathrm{Using}\;\mathrm{AdT}:\;\;K_{\mathrm{oxz}}^{\mathrm{ab}}={\left(\frac{1}{K_{\mathrm{bs}}}+\frac{1}{K_{m}^{\mathrm{ab}}}\right)}^{-1}=\frac{E^{\mathrm{ab}}+(E^{\mathrm{ad}}≅0)}{\psi_{\mathrm{AQD}}^{\mathrm{xyl}}-\psi_{\mathrm{AQD}}^{\mathrm{ab}}};$$

The values collected with the abaxial side taped (Figs. 1, A and D(iii), 4, B and E), were used to interrogate the adaxial side:


(4) $$\;\mathrm{Using}\;\mathrm{AbT}:\;\;K_{\mathrm{oxz}}^{\mathrm{ad}}={\left(\frac{1}{K_{\mathrm{bs}}}+\frac{1}{K_{m}^{\mathrm{ad}}}\right)}^{-1}=\frac{(E^{\mathrm{ab}}≅0)+E^{\mathrm{ad}}}{\psi_{\mathrm{AQD}}^{\mathrm{xyl}}-\psi_{\mathrm{AQD}}^{\mathrm{ab}}}$$

The contributions of bundle-sheath and mesophyll to the total conductance were evaluated using circuit analogy as shown in Figs. 1, A and D(ii), (iii), 4D and E, with $K_{\mathrm{bs}\;}$ calculated as follows:


(5) $$\;\mathrm{Using}\;\mathrm{AdT}:\;\;K_{\mathrm{bs}}(\mathrm{AdT})=\frac{E^{\mathrm{ab}}+(E^{\mathrm{ad}}≅0)}{\psi_{\mathrm{AQD}}^{\mathrm{xyl}}-\psi_{\mathrm{AQD}}^{\mathrm{ad}}},\;\mathrm{and}\;$$

(6) $$\;\mathrm{Using}\;\mathrm{AbT}:\;\;K_{\mathrm{bs}}(\mathrm{AbT})=\frac{(E^{\mathrm{ab}}≅0)+E^{\mathrm{ad}}}{\psi_{\mathrm{AQD}}^{\mathrm{xyl}}-\psi_{\mathrm{AQD}}^{\mathrm{ab}}}$$

and mesophyll conductances of tissue between xylem and adaxial surface $\left(K_{m}^{\mathrm{ad}}\right)$, and xylem and abaxial surface $\left(K_{m}^{\mathrm{ab}}\right)$ were calculated as follows:


(7) $$\mathrm{Using}\;\mathrm{AdT}:\;K_{m}^{\mathrm{ab}}=\frac{E^{\mathrm{ab}}+(E^{\mathrm{ad}}≅0)}{\psi_{\mathrm{AQD}}^{\mathrm{ad}}-\psi_{\mathrm{AQD}}^{\mathrm{ab}}},\;\mathrm{and}$$

(8) $$\;\mathrm{Using}\;\mathrm{AbT}:\;\;K_{m}^{\mathrm{ad}}=\frac{(E^{\mathrm{ab}}≅0)+E^{\mathrm{ad}}}{\psi_{\mathrm{AQD}}^{\mathrm{ab}}-\psi_{\mathrm{AQD}}^{\mathrm{ad}}}$$

All the conductances as a function of decreasing xylem water potentials (Fig. 4G to I) were fit with a sigmoidal form (Scoffoni et al. 2017) as follows:


(9) $$K(\psi )=K^{\max}/\left(1+{\left(\frac{\psi }{\psi_{50\text{\%}}}\right)}^{p}\right)$$

The parameters that yielded the best-fit solution are reported in Supplementary Tables S4, S5, and S6.

**Table 1.** Definitions for acronyms

Abbreviation	Definition
*ψ*	Water potential
$\psi^{\mathrm{stem}\;}$	Stem water potential
$\psi^{\mathrm{xyl}}$	Xylem water potential
$\psi_{\mathrm{AQD}}^{\mathrm{xyl}}$	Xylem water potential measured using AquaDust at a given location along the length of the leaf
$\psi^{\mathrm{bs}}$	Water potential at bundle sheath-mesophyll/air interface
$\psi^{\mathrm{term}\text{-}\mathrm{ad}\;}$	Water potential corresponding to terminal evaporating surfaces of mesophyll adjacent to stomata toward adaxial (ad) leaf surface
$\psi^{\mathrm{term}\text{-}\mathrm{ab}\;}$	Water potential corresponding to terminal evaporating surfaces of mesophyll adjacent to stomata toward abaxial (ab) leaf surface
$\psi_{\mathrm{SPC}}^{\mathrm{leaf}\;}$	Tissue-average water potential of leaf tissue measured using SPC
BT	Both sides taped
NT	No sides taped
AdT	Adaxial surface taped
AbT	Abaxial surface taped
$\psi_{\mathrm{AQD}}^{\mathrm{BT}-\mathrm{ad}}$	Water potential corresponding to terminal evaporating surfaces of mesophyll adjacent to stomata toward adaxial (ad) leaf surface taped on both sides (BT)
$\psi_{\mathrm{AQD}}^{\mathrm{BT}-\mathrm{ab}}$	Water potential corresponding to terminal evaporating surfaces of mesophyll adjacent to stomata toward abaxial (ab) leaf surface taped on both sides (BT)
$\psi_{\mathrm{AQD}}^{\mathrm{NT}-\mathrm{ad}}$	Water potential corresponding to terminal evaporating surfaces of mesophyll adjacent to stomata toward adaxial (ad) leaf surface not taped on any sides (NT)
$\psi_{\mathrm{AQD}}^{\mathrm{BT}-\mathrm{ab}}$	Water potential corresponding to terminal evaporating surfaces of mesophyll adjacent to stomata toward abaxial (ab) leaf surface not taped on any sides (NT)
$\psi_{\mathrm{AQD}}^{\mathrm{AdT}-\mathrm{ad}}$	Water potential corresponding to terminal evaporating surfaces of mesophyll adjacent to stomata toward adaxial (ad) leaf surface with AdT
$\psi_{\mathrm{AQD}}^{\mathrm{AdT}-\mathrm{ab}}$	Water potential corresponding to terminal evaporating surfaces of mesophyll adjacent to stomata toward abaxial (ab) leaf surface with AdT
$\psi_{\mathrm{AQD}}^{\mathrm{AbT}-\mathrm{ad}}$	Water potential corresponding to terminal evaporating surfaces of mesophyll adjacent to stomata toward adaxial (ad) leaf surface with AbT
$\psi_{\mathrm{AQD}}^{\mathrm{AbT}-\mathrm{ab}}$	Water potential corresponding to terminal evaporating surfaces of mesophyll adjacent to stomata toward abaxial (ab) leaf surface with AbT
$K_{\mathrm{xyl}}$	Conductance of the xylem tissue
$K_{\mathrm{oxz}}^{\mathrm{SPC}}$	Conductance of the OXZ defined by water potentials measured using SPC (see Methods—Eq. 1)
$K_{\mathrm{oxz}}^{\mathrm{ab}}$	Conductance of the OXZ toward abaxial surface defined by water potentials measured using AquaDust (Fig. 1A, see Methods—Eq. 3)
$K_{\mathrm{oxz}}^{\mathrm{ad}}$	Conductance of the OXZ toward adaxial surface defined by water potentials measured using AquaDust (Fig. 1A, see Methods—Eq. 4)
$K_{\mathrm{bs}}$	Conductance of the bundle-sheath tissue (see Methods, Eqs. 5 and 6)
$K_{m}^{\mathrm{ab}}$	Conductance of the abaxial mesophyll tissue (Fig. 1A, see Methods, Eq. 7)
$K_{m}^{\mathrm{ad}}$	Conductance of the adaxial mesophyll tissue (Fig. 1A, see Methods, Eq. 8)
*E*	Transpiration rate $\left(\mathrm{mmol}/m^{2}/s\right)$
*A*	Assimilation rate $\left(\mu \mathrm{mol}/m^{2}/s\right)$
$g_{s}$	Stomatal conductance $\left(\mathrm{mmol}/m^{2}/s\right)$

## Supplementary Material

kiad679_Supplementary_Data

## Data Availability

The data underlying this article are available in the article and in its online supplementary material.

## Supplementary data

The following materials are available in the online version of this article.

**Supplementary Methods S1.** Design and preparation of AquaDust reporters.

**Supplementary Methods S2.** AquaDust injection into leaves.

**Supplementary Methods S3.** Measuring local water potential with an optical point probe.

**Supplementary Methods S4.** Sample preparation for cryogenic scanning electron micrographs in Fig. 2A—main text.

**Supplementary Figure S1.** AquaDust measurements with controlled transpiration along the length of the maize leaf.

**Supplementary Figure S2.** Hydraulic model for a maize leaf with four treatments as described in Fig. 4—main text.

**Supplementary Figure S3.** Prediction of water potential distribution throughout maize leaf cross-section as predicted using finite-element model.

**Supplementary Figure S4.** Hydraulic resistance corresponding to Fig. 4, G to I—main text.

**Supplementary Table S1.** Quantitative values and analysis of gas exchange measurements presented in Fig. 4B.

**Supplementary Table S2.** Coefficients (with 95% confidence intervals) corresponding to linear fit (*y* = −1/*K* × *x*) and translucent bands in Fig. 4F.I—main text.

**Supplementary Table S3.** Coefficients (with 95% confidence intervals) corresponding to linear fit (*y* = −1/*K* × *x*) and translucent bands in Fig. 4F.II—main text.

**Supplementary Table S4.** Coefficients (with 95% confidence intervals) corresponding to vulnerability curves in Fig. 4G—main text.

**Supplementary Table S5.** Coefficients (with 95% confidence intervals) corresponding to bundle-sheath vulnerability curves in Fig. 4H—main text.

**Supplementary Table S6.** Coefficients (with 95% confidence intervals) corresponding to mesophyll vulnerability curves in Fig. 4I—main text.

**Supplementary Movie S1.** Confocal *z*-stack micrograph of a maize (*Zea Mays L.*) leaf shows AquaDust distribution within mesophyll.

## Dive Curated Terms

The following phenotypic, genotypic, and functional terms are of significance to the work described in this paper:

water CHEBI: CHEBI:15377
