# Supplementary material for: Localized measurements of water potential reveal large loss of conductance in living tissues of maize leaves
Source: Plant Physiol. 2023 Dec 21;194(4):2288–300. doi: 10.1093/plphys/kiad679 (PMC10980393; doi:10.1093/plphys/kiad679)
Supplement: kiad679_Supplementary_Data [file kiad679_supplementary_data.zip › Supplemental Data.pdf]

Supplementary Information for

Localized measurements of water potential reveal large loss of conductance in living tissues of maize leaves

Piyush Jain, Annika E. Huber, Fulton E. Rockwell, Sabyasachi Sen, N. Michele Holbrook, Abraham D. Stroock

Corresponding Author: Piyush Jain, Abraham D. Stroock

E-mail: pj248@cornell.edu, abe.stroock@cornell.edu

This PDF file includes:

Contents

S1 Design and preparation of AquaDust reporters **3**

S2 AquaDust injection into leaves **3**

S3 Measuring local water potential with an optical point probe **3**

S4 Sample preparation for cryogenic scanning electron micrographs in Fig. 2 A - main text **4**

List of Figures

S1 AquaDust measurements with controlled transpiration along the length of the maize leaf. 5

S2 Hydraulic model for a maize leaf with four treatments as described in Fig. 4-main text 6

S3 Prediction of water potential distribution throughout maize leaf cross-section as predicted using finite-element model 6

S4 Hydraulic resistance corresponding to Fig. 4G-I - main text 7

List of Tables

S1 Quantitative values and analysis of gas exchange measurements presented in Fig. 4B 8

S2 Coefficients (with 95% confidence intervals) corresponding to linear fit ($y = -\frac{1}{K} \times x$) and translucent bands in Fig. 4F.I - main text 8
S3 Coefficients (with 95% confidence intervals) corresponding to linear fit ($y = -\frac{1}{K} \times x$) and translucent bands in Fig. 4F.II - main text 8
S4 Coefficients (with 95% confidence intervals) corresponding to vulnerability curves in Fig. 4G - main text 9
S5 Coefficients (with 95% confidence intervals) corresponding to bundle-sheath vulnerability curves in Fig. 4H - main text 9
S6 Coefficients (with 95% confidence intervals) corresponding to mesophyll vulnerability curves in Fig. 4I - main text 9

S1. Design and preparation of AquaDust reporters

We use AquaDust as a nano-scale probe of water potential. AquaDust particles are nanometer-scale hydrogel spheres with FRET-paired fluorophores incorporated into the polymer network (Fig. 1B - main text). Synthesis, characterization, and calibration of AquaDust are described in detail previously (Jain et al., 2021). In the FRET (Förster Resonant Energy Transfer) process, excitation of one fluorophore (the donor dye) leads to energy transfer to the second fluorophore (acceptor dye) with an efficiency proportional to the distance between the donor (green – Fig. 1B) and acceptor (yellow – Fig. 1B) dyes. Here, we have covalently linked FRET pairs into a polyacrylamide network, resulting in 30-100 nm diameter hydrogel particles. Changes in the water potential within the tissue in which AquaDust is suspended result in the shrinking or swelling of the gel, which changes the efficiency of energy transfer (FRET) between the two dyes. These changes in efficiency are detected as changes in the relative intensity of the emission peaks of the donor and acceptor dyes in the fluorescence spectrum of the leaf (Jain et al., 2021). Here, we collected FRET measurements with a spectrometer on intact leaves infiltrated using appropriate filters for excitation and emission light on the adaxial and abaxial sides of the leaf.

S2. AquaDust injection into leaves

AquaDust (20 μl per injection) was injected into maize leaves through a syringe that was gently pressed against the leaves one day before measurement to allow the aqueous buffer, in which AquaDust was dissolved, time to transpire. Successful injections were characterized by a dark green colorization of the leaf tissue (approximately 1-2 cm^2) caused by AquaDust suspension filling the mesophyll. To avoid AquaDust extrusion out of open stomata during injection, plants were removed from the growth chamber and placed in the shade to invoke stomatal closure. Leaf surfaces were gently scratched with a razor blade ($\sim$ 2 mm) around the injection site (for further characterization, see (Jain et al., 2021)). After AquaDust injection, leaves were rinsed off with water to clean the leaf from AquaDust on the leaf surface. Then, plants were returned to the growth chamber and left for >24 hours before measurements were taken. To exclude non-meaningful water potential data caused by potential tissue damage during injection, local leaf water potential measurements with AquaDust have been performed at least 3 mm away from the injection site (for further characterization, see (Jain et al., 2021)).

S3. Measuring local water potential with an optical point probe

Local water potentials in the mesophyll were measured by recording the FRET intensity of AquaDust which then was converted into water potential values. Signal recording and conversion is described earlier (Jain et al., 2021). Briefly, AquaDust FRET intensity was collected with a fiber optic

point probe (QR600-7-UV-125F, Premium 600-micron Reflection Probe, Ocean Optics Inc.), by clamping onto the leaf. Excitation light was emitted by a mercury lamp light (Leica EL6000) with a narrow-band optical filter (470 nm-500 nm) and directed through the point probe fitted in a collimator (74 VIS Fiber Optic Collimator, Ocean Optics Inc.) and attached to an opaque leaf clamp. The leaf clamp shielded measurements from scattered environmental light. The reflected light captured by the reflection probe was passed through a long-pass cutoff filter (> 510 nm) and transmitted to a spectrometer (Ocean Optics Inc., ST2000, Dunedin, Fl). The spectra were saved using OceanView software operating with an integration time of 2-4 sec averaged 3 times.

We recorded between three and six spectra from one infiltration site by moving the optical probe across the infiltration zone and calculated the mean FRET by averaging over the multiple spectra. No distinction was made in the data collected from the upstream/downstream of the AquaDust-infiltration site. The fluorescence spectra from the leaf not infiltrated with AquaDust were saved as background signals and subtracted from the spectra collected from AquaDust-infiltrated leaves.

For calculating experimental FRET Efficiency, we determined the relative contributions of the donor and acceptor emissions (x and y) by spectrally decomposing the emission spectra, Em_{exp}:

$$\begin{bmatrix} Em_{\mathrm{Donor}} & Em_{\mathrm{Acceptor}} \end{bmatrix} \begin{bmatrix} x \\ y \end{bmatrix} = Em_{\mathrm{exp}} \qquad \text{[S1]}$$

where Em_{Donor} and Em_{Acceptor} are the normalized emission spectra of the donor and acceptor as supplied by the chemical vendor. We performed this decomposition using 'lsqnonneg' solver in the MATLAB software.

Experimental relative FRET Efficiency was calculated as the following ratio:

$$\zeta_{\mathrm{exp}} = \frac{y}{x+y} \qquad \text{[S2]}$$

S4. Sample preparation for cryogenic scanning electron micrographs in Fig. 2 A - main text

We collected cryogenic scanning electron images by freezing leaves with a liquid nitrogen pre-cooled copper clamp while still attached to the plants and stored in liquid nitrogen. The sample was sputter-coated with platinum at -130°C with a MED020 Coating System (Bal Tec) and imaged using an accelerating voltage of 3 kV in an NVision 40 Dual-Beam focused ion beam and scanning electron microscope (Zeiss) with a pixel resolution of 395 nm.

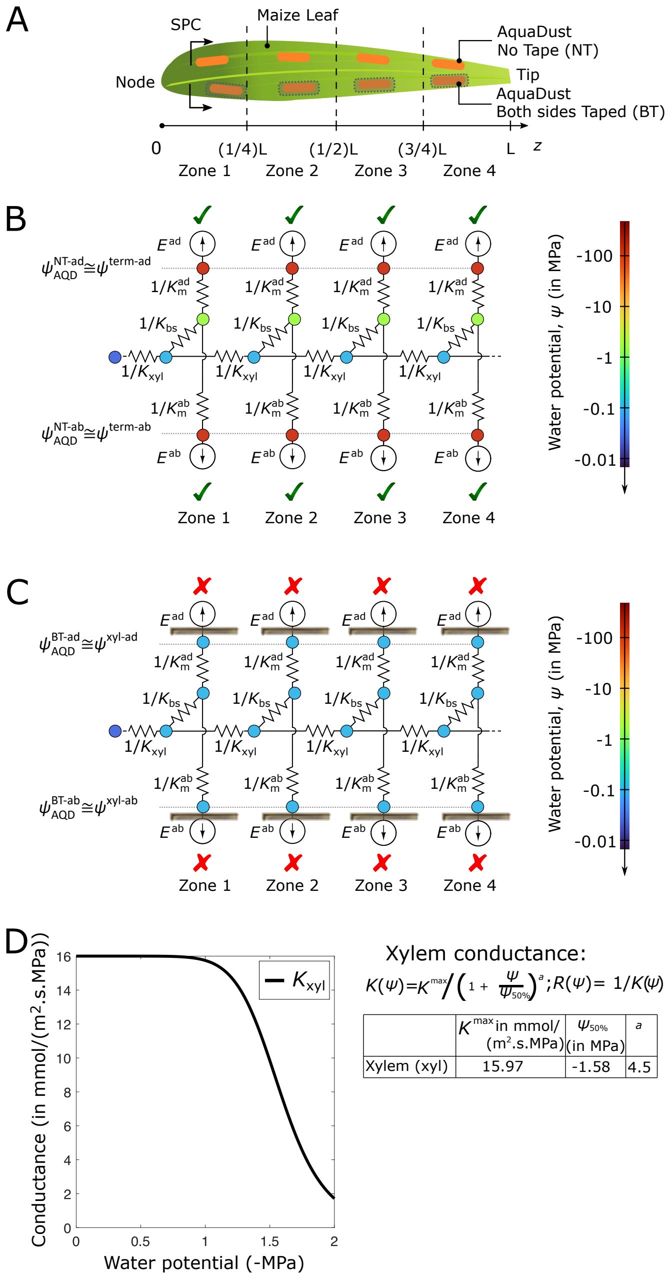

Fig. S1. AquaDust measurements with controlled transpiration along the length of the maize leaf. (A) Diagram of maize leaf with eight regions infiltrated with AquaDust (orange) and either without any tape on either side (not-taped -NT) or covered on both sides with impermeable tape (BT). SPC measurements were performed on cut sections from the positions indicated to the tip. (B) Diagram of a hypothetical hydraulic circuit model of leaf cross-section with four zones that correspond to the no-taped (NT) sites of measurements in (A). In each segment, we show the expected gradient in water potential resulting from the conductance both in the xylem (K_{xyl}) and outside the xylem, comprising of bundle-sheath (K_{bs}) and mesophyll towards adaxial surface ($K_{\mathrm{m}}^{\mathrm{ad}}$) and abaxial surface ($K_{\mathrm{m}}^{\mathrm{ab}}$) along with the transpiration rate from adaxial (E^{ad}) and abaxial surface (E^{ab}). The measurements of water potential with AquaDust ($\psi_{\mathrm{AQD}}^{\mathrm{NT-ad(b)}}$) are assumed to correspond to the water potential of the terminal evaporating surfaces ($\psi^{\mathrm{term-ad(b)}}$) in each segment. (C) Diagram of a hypothetical hydraulic circuit model of leaf cross-section with four zones that correspond to both sides-taped (BT) sites of measurements in (A). Blocking of transpiration from both sides of the leaf causes the tissue to be in local equilibrium with xylem water potential such that measurements of water potential with AquaDust ($\psi_{\mathrm{AQD}}^{\mathrm{BT-ad(b)}}$) are assumed to correspond to the xylem water potential ($\psi^{\mathrm{xyl-ad(b)}}$) in each segment. (D) Logistic function fit for xylem conductance, K_{xyl} to the vulnerability curve for xylem obtained by Li et al. (Li et al., 2009).

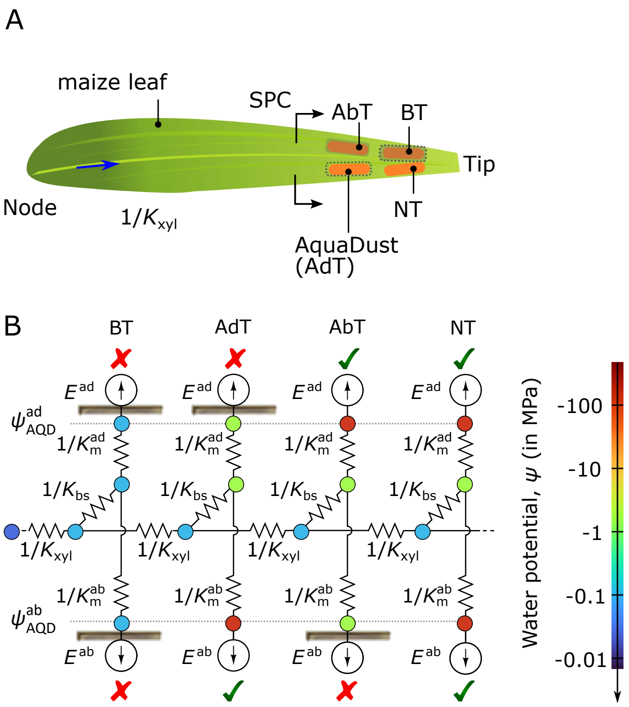

Fig. S2. Hydraulic model for a maize leaf with four treatments as described in Fig. 4-main text. (A) Diagram of maize leaf with four regions infiltrated with AquaDust (orange) suppressing transpiration (E) by applying tape on both sides (BT), applying tape on adaxial surface (AdT), applying tape on abaxial surface (AbT), and a control region with no tape (NT). SPC measurements were performed on cut sections from the positions indicated to the tip. (B) Diagram of a hypothetical hydraulic circuit model of leaf cross-section with four cases (BT, AdT, AbT, and NT) that correspond to the sites of measurements in (A). In each segment, we show expected gradient in water potential resulting from the conductance both in the xylem (K_{xyl}) and outside the xylem, comprising of bundle-sheath (K_{bs}) and mesophyll towards adaxial surface ($K_{\mathrm{m}}^{\mathrm{ad}}$) and abaxial surface ($K_{\mathrm{m}}^{\mathrm{ab}}$) along with the transpiration rate from adaxial (E^{ad}) and abaxial surface (E^{ab}). The measurements of water potential with AquaDust are assumed to correspond to water potential below the adaxial epidermis ($\psi_{\mathrm{AQD}}^{\mathrm{ad}}$) and abaxial epidermis ($\psi_{\mathrm{AQD}}^{\mathrm{ab}}$) in each segment.

.

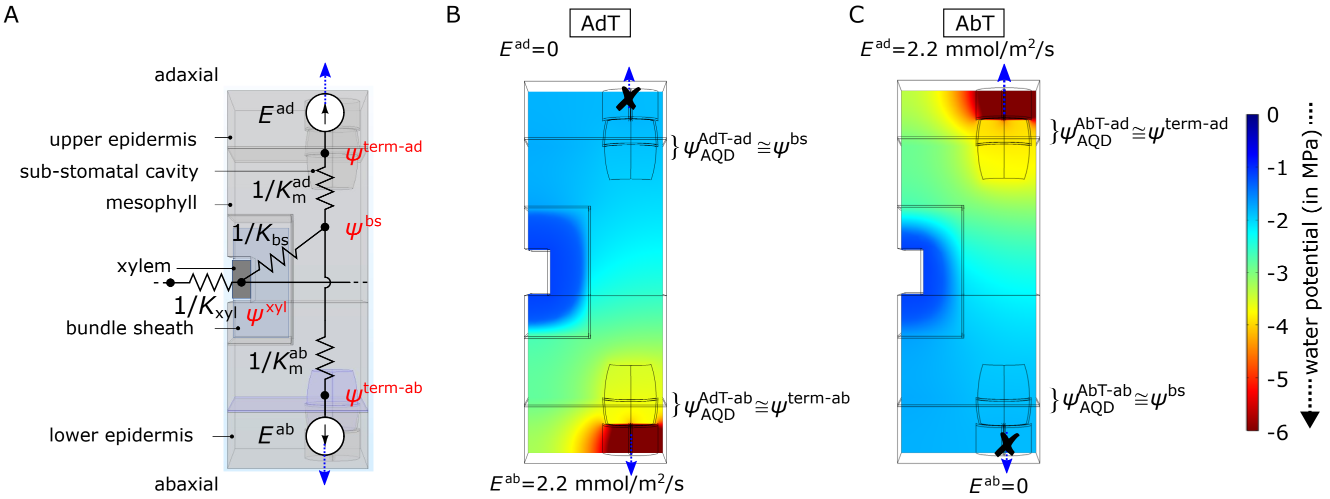

Fig. S3. Prediction of water potential distribution throughout maize leaf cross-section as predicted using finite-element model: (A) Domain for finite-element solution, showing various compartments in the model namely upper epidermis, lower epidermis, sub-stomatal cavity, mesophyll, bundle-sheath and xylem (see Rockwell et al. (Rockwell et al., 2014a,b) for details of the model) with a solution for distribution of ψ in a leaf cross-section with $\psi_{\mathrm{AQD}}^{\mathrm{xyl}}$ of -0.5 MPa, with: (B) adaxial surface taped (AdT) and (C) abaxial surface taped (AbT), where $E^{\mathrm{ad(b)}} = 2.2$ mmol/m^2/s.

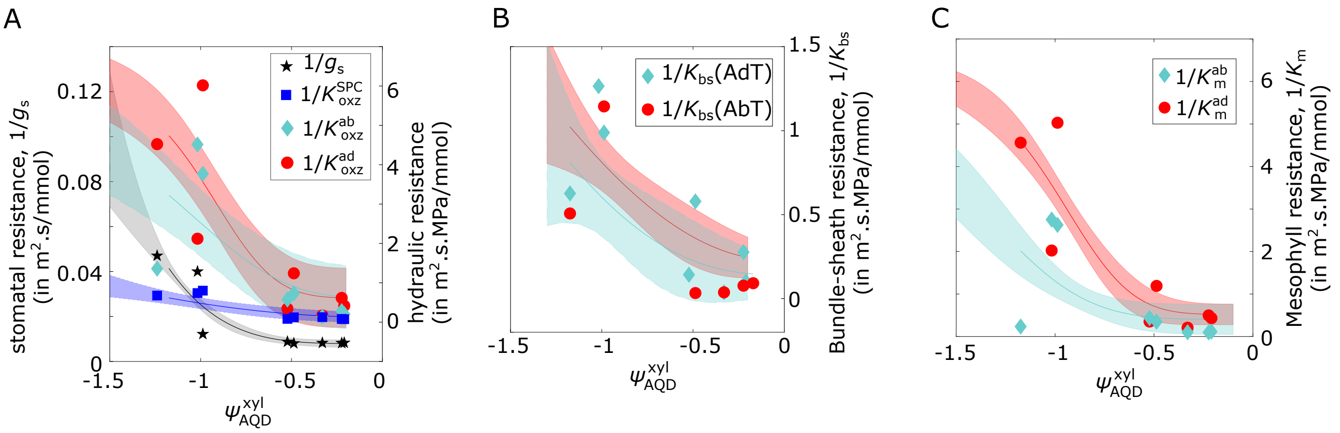

Fig. S4. Hydraulic resistance corresponding to Fig. 4G-I - main text: (A) Variation of stomatal resistance ($1/g_s$ - black stars), effective leaf resistance ($1/K_{\rm oxz}^{\rm SPC}$ – squares – Eq. S3), adaxial and abaxial outside-xylem resistance ($1/K_{\rm oxz}^{\rm ad}$ and $1/K_{\rm oxz}^{\rm ab}$ – circles and diamonds – Eqs. S5 and S6) as a function of xylem potential based on AquaDust measurement in BT region ($\psi_{\rm AQD}^{\rm xyl}$). (B-C) Variation of bundle-sheath resistance $1/K_{\rm bs}$ (B) and of $1/K_{\rm m}^{\rm ad}$ and $1/K_{\rm m}^{\rm ab}$ (C), calculated for the AdT and AbT zones as a function of $\psi_{\rm AQD}^{\rm xyl}$.

Table S1. Quantitative values and analysis of gas exchange measurements presented in Fig. 4B

Values (Mean±S.E.) corresponding to data shown in Fig. 4C

	BT	AdT	AbT	NT
Assimilation Rate (A)	1.3 ± 0.6	17.4 ± 2.9	12.7 ± 4.0	14.8 ± 3.8
Transpiration Rate (E)	0.32 ± 0.26	2.55 ± 0.32	2.14 ± 0.56	2.50 ± 0.52

p-value from two sample equal variance two-tailed t-test for $n = 4$ replicates corresponding to A

	BT	AdT	AbT	NT
BT		0.002	0.034	0.008
AdT			0.170	0.395
AbT				0.538

p-value from two sample equal variance two-tailed t-test for $n = 4$ replicates corresponding to E

	BT	AdT	AbT	NT
BT		0.004	0.033	0.012
AdT			0.333	0.907
AbT				0.451

p-values<0.05 are shown in red.

Table S2. Coefficients (with 95% confidence intervals) corresponding to linear fit ($y = -\frac{1}{K} \times x$) and translucent bands in Fig. 4F.I - main text

Data to be fitted	K	R^2	rmse
x-axis: $\Delta\psi = \psi^{\mathrm{leaf}}_{\mathrm{SPC}} - \psi^{\mathrm{xyl}}_{\mathrm{AQD}}$, y-axis: E^{ab} (blue squares)	161.7 (13.5, -16.4)	0.052	0.125
x-axis: $\Delta\psi = \psi^{\mathrm{ad}}_{\mathrm{AQD}} - \psi^{\mathrm{xyl}}_{\mathrm{AQD}}$, y-axis: E^{ab} (red circles)	14.0 (8.8, -3.9)	0.130	0.147
x-axis: $\Delta\psi = \psi^{\mathrm{ab}}_{\mathrm{AQD}} - \psi^{\mathrm{xyl}}_{\mathrm{AQD}}$, y-axis: E^{ab} (cyan diamonds)	2.7 (1.4, 3.3)	0.941	0.118

Table S3. Coefficients (with 95% confidence intervals) corresponding to linear fit ($y = -\frac{1}{K} \times x$) and translucent bands in Fig. 4F.II - main text

Data to be fitted	K	R^2	rmse
x-axis: $\Delta\psi = \psi^{\mathrm{leaf}}_{\mathrm{SPC}} - \psi^{\mathrm{xyl}}_{\mathrm{AQD}}$, y-axis: E^{ad} (blue squares)	116.6 (11.2, -13.1)	0.050	0.125
x-axis: $\Delta\psi = \psi^{\mathrm{ad}}_{\mathrm{AQD}} - \psi^{\mathrm{xyl}}_{\mathrm{AQD}}$, y-axis: E^{ad} (red circles)	2.02 (1.34, 4.1)	0.581	0.378
x-axis: $\Delta\psi = \psi^{\mathrm{ab}}_{\mathrm{AQD}} - \psi^{\mathrm{xyl}}_{\mathrm{AQD}}$, y-axis: E^{ad} (cyan diamonds)	-8.99 (48.5, -4.1)	0.193	0.201

Table S4. Coefficients (with 95% confidence intervals) corresponding to vulnerability curves in Fig. 4G - main text

Conductance, $K(\psi) = k^{\max}/\left(1 + \left(\frac{\psi}{\psi_{50\%}}\right)^p\right)$	$K^{\max}$ (in $\mathrm{mmol/m^2.s.MPa}$)	$\psi_{50\%}$ (in MPa)	p
$K_{\mathrm{oxz}}^{\mathrm{SPC}}$ vs. $\psi_{\mathrm{AQD}}^{\mathrm{xyl}}$	15.2 (11.5, 17.8)	-0.7 (-0.6, -0.9)	1.4 (1.1, 2.1)
$K_{\mathrm{oxz}}^{\mathrm{ab}}$ vs. $\psi_{\mathrm{AQD}}^{\mathrm{xyl}}$	4.6 (3.5, 7.1)	-0.6 (-0.5, -0.7)	4.1 (3.2, 8.2)
$K_{\mathrm{oxz}}^{\mathrm{ad}}$ vs. $\psi_{\mathrm{AQD}}^{\mathrm{xyl}}$	3.1 (1.8, 4.3)	-0.6 (-0.3, -0.8)	5.9 (1.9, 9.8)
Stomatal Conductance, $g_{\mathrm{s}}(\psi) = g_{\mathrm{s}}^{\max}/\left(1 + \left(\frac{\psi}{\psi_{50\%}}\right)^p\right)$	$g_{\mathrm{s}}^{\max}$ (in $\mathrm{mmol/m^2.s}$)	$\psi_{50\%}$ (in MPa)	p
g_{s} vs. $\psi_{\mathrm{SPC}}^{\mathrm{stem}}$	120.4 (102.1, 138.8)	-1.0 (-1.1, -0.9)	11.2 (10.8, 12.3)

Table S5. Coefficients (with 95% confidence intervals) corresponding to bundle-sheath vulnerability curves in Fig. 4H - main text

Conductance, $K(\psi) = k^{\max}/\left(1 + \left(\frac{\psi}{\psi_{50\%}}\right)^p\right)$	$K^{\max}$ (in $\mathrm{mmol/m^2.s.MPa}$)	$\psi_{50\%}$ (in MPa)	p
$K_{\mathrm{bs}}(\mathrm{AdT})$ vs. $\psi_{\mathrm{AQD}}^{\mathrm{xyl}}$	11.4 (9.1, 15.7)	-0.6 (-0.6, -0.7)	5.3 (4.9, 5.8)
$K_{\mathrm{bs}}(\mathrm{AbT})$ vs. $\psi_{\mathrm{AQD}}^{\mathrm{xyl}}$	20.3 (12.0, 22.6)	-0.7 (-0.6, -0.8)	9.4 (7.7, 10.9)

Table S6. Coefficients (with 95% confidence intervals) corresponding to mesophyll vulnerability curves in Fig. 4I - main text

Conductance, $K(\psi) = k^{\max}/\left(1 + \left(\frac{\psi}{\psi_{50\%}}\right)^p\right)$	$K^{\max}$ (in $\mathrm{mmol/m^2.s.MPa}$)	$\psi_{50\%}$ (in MPa)	p
$K_{\mathrm{m}}^{\mathrm{ab}}$ vs. $\psi_{\mathrm{AQD}}^{\mathrm{xyl}}$	8.7 (6.9, 10.4)	-0.6 (-0.5, -0.7)	4.5(4.7, 5.1)
$K_{\mathrm{m}}^{\mathrm{ad}}$ vs. $\psi_{\mathrm{AQD}}^{\mathrm{xyl}}$	2.8 (1.9, 4.5)	-0.5 (-0.5, -0.6)	5.9 (4.7, 6.8)

References

Jain P, Liu W, Zhu S, Melkonian J, Pauli D, Riha SJ, Gore M, Stroock A. 2021. A minimally disruptive method for measuring water potential in-planta using hydrogel nanoreporters. Proceedings of the National Academy of Sciences of the United States of America :1–9

Li Y, Sperry JS, Shao M, North GB, Maharaj FDR, Phillips CA, Lynch FH, Woodside WT. 2009. Hydraulic conductance and vulnerability to cavitation in corn (Zea mays L.) hybrids of differing drought resistance. Frontiers in Plant Science 66:341–346.

Rockwell FE, Holbrook NM, Stroock AD. 2014a. The competition between liquid and vapor transport in transpiring leaves. Plant Physiology 164:1741–1758.

Rockwell FE, Michele Holbrook N, Stroock AD. 2014b. Leaf hydraulics I: Scaling transport properties from single cells to tissues. Journal of Theoretical Biology 340:251–266.
