# Supplementary material for: Localized measurements of water potential reveal large loss of conductance in living tissues of maize leaves
Source: Plant Physiol. 2023 Dec 21;194(4):2288–300. doi: 10.1093/plphys/kiad679 (PMC10980393; doi:10.1093/plphys/kiad679)
Supplement: kiad679_Supplementary_Data [file kiad679_supplementary_data.zip › Supplemental Movie Legends.pdf]

Supplemental Movie S1: Confocal z-stack micrograph of a maize (*Zea Mays* L.) leaf shows AquaDust distribution within mesophyll: Video shows fluorescence emission from AquaDust false colored as yellow and fluorescence emission from chlorophyll false-colored as red. A subsequent video shows the native fluorescence from a control leaf without AquaDust for comparison. See Materials and Methods for details of preparation and imaging.
